# In this month’s Bulletin

**DOI:** 10.2471/BLT.19.001119

**Published:** 2019-11-01

In an editorial, Ab Fatah Ab Rahman (729) argues for increased capacity to monitor adverse drug reactions in community settings. 

In the news section, Sophie Cousins (733–734) reports on urban programmes to reduce gun violence in Colombia and interviews Balram Bhargava (735–736) about his efforts to foster needed innovation in the health sector.

## Burkina Faso

### Provision of family planning services

Tieba Millogo et al. (783–788) examine the effects of task-shifting.

## Malawi, Zimbabwe

### Informing female sex workers of their HIV status 

Sue Napierala et al. (764–776) study ways of providing self-testing kits.

## Russian Federation

### East of the Urals

Ivan Meshkov et al. (737–745) examine the variables affecting tuberculosis prevalence.

## United Kingdom of Great Britain and Northern Ireland

### Measles vaccination coverage

Michael Edelstein et al. (754–763) triangulate data sources.

## Global

### Paying for universal health coverage 

Bernadette Ann-Marie O’Hare (746–753) estimates the potential health sector gains of addressing international corporate tax avoidance.

### Physical fitness of young people

Neil Armstrong and Jo Welsman (777–782) examine myths and misconceptions.

